# Infection History and Current Coinfection With *Schistosoma mansoni* Decreases *Plasmodium* Species Intensities in Preschool Children in Uganda

**DOI:** 10.1093/infdis/jiac072

**Published:** 2022-03-05

**Authors:** Daniel McDowell, Lisa Hurt, Narcis B Kabatereine, John Russell Stothard, Joanne Lello

**Affiliations:** School of Biosciences, Cardiff University, Cardiff, United Kingdom; School of Medicine, Cardiff University, Cardiff, United Kingdom; Vector Control Division, Ministry of Health, Kampala, Uganda; Department of Tropical Disease Biology, Liverpool School of Tropical Medicine, Liverpool, United Kingdom; School of Biosciences, Cardiff University, Cardiff, United Kingdom

**Keywords:** coinfection, *Schistosoma mansoni*, *Plasmodium* species, preschool-aged children, prior infections

## Abstract

Malaria–schistosomiasis coinfections are common in sub-Saharan Africa but studies present equivocal results regarding the interspecific relationships between these parasites. Through mixed-model analyses of a dataset of Ugandan preschool children, we explore how current coinfection and prior infection with either *Schistosoma mansoni* or *Plasmodium* species alter subsequent *Plasmodium* intensity, *Plasmodium* risk, and *S mansoni* risk. Coinfection and prior infections with *S mansoni* were associated with reduced *Plasmodium* intensity, moderated by prior *Plasmodium* infections, wealth, and host age. Future work should assess whether these interactions impact host health and parasite control efficacy in this vulnerable age group.

## Teaser

*Plasmodium-Schistosoma mansoni*coinfections are associated with a reduced *Plasmodium*intensity in preschool-aged children. This relationship, however, is modified by prior infections, host age, and family wealth.

Malaria and schistosomiasis are 2 of the most important tropical parasitic diseases, each infecting >200 million people per year with substantial morbidity and mortality [1, 2]. Preschool-aged children (<6 years of age) can be at high risk for both infections [1, 2]. Throughout sub-Saharan Africa, malaria and schistosomiasis are coendemic, and coinfections (ie, both species infecting the host simultaneously) are common [3]. Several research studies suggest that *Plasmodium* species and schistosomes interact during coinfection, yet none have explicitly considered the consequences of coinfection in preschool-aged children. This exposes a crucial knowledge gap in current options for effective control in younger children with no combined control strategy currently developed [1, 2].

Prior studies of interactions between *Plasmodium* and schistosomes have provided equivocal outcomes, with some studies reporting increased *Plasmodium* intensity, prevalence, or incidence when in coinfection with schistosomes [3–5], while others suggest a decrease [3, 6]. All studies to date have been conducted on older cohorts. Older study cohorts are likely to have had multiple cycles of infection and treatment and to have developed acquired immunity for both *Plasmodium* and schistosomes, biasing the immune system in ways that may contribute to disparities observed between studies [7].

To shed light on the dynamics of malaria and schistosomiasis in young children, we examined *Plasmodium*–*Schistosoma mansoni* coinfections in preschool-aged children and assessed whether prior infections and coinfections altered *S mansoni* and *Plasmodium* prevalence and *Plasmodium* intensity. We conducted an in-depth secondary analysis of the Schistosomiasis in Mothers and Infants (SIMI) dataset [8], which contains infection histories for each child, enabling us to tease apart the relationship between prior infections and current coinfections for *Plasmodium* and *S mansoni*. Previous work indicates that *Plasmodium falciparum* is the main species in our study regions [9]. The SIMI dataset does not, however, include species-specific measures and, therefore, we will use *Plasmodium* throughout since we cannot be certain of the *Plasmodium* species. In line with studies in older children, we hypothesize that *S mansoni* will increase the risk and intensity of *Plasmodium* infections but that *S mansoni* will be unaffected by *Plasmodium*.

## MATERIALS AND METHODS

The SIMI project was approved by the London School of Hygiene and Tropical Medicine, United Kingdom (LSHTM 5538.09), and the Ugandan National Council of Science and Technology. In brief, for primary data collection informed consent was provided in writing, or by a thumbprint in cases of illiteracy, by mothers for themselves and on behalf of their children before enrollment (see further details in [8]).

The SIMI study took place in Uganda at 6 villages around Lake Albert (Piida, Bugoigo, Walukuba) and Lake Victoria (Bugoto, Bukoba, Lwanika), where infections in each child and mother were assessed at 6-month intervals. We used data from the initial baseline assessment and the 6-month follow-up due to participant dropout at later surveys. Extracted data detail *S mansoni* and *Plasmodium* infections of preschool-aged children, presence/absence of any soil-transmitted helminths (STHs), information on infection risk behaviors, age, sex, tribe, and mother’s education level and occupation. Variables indicative of a family’s socioeconomic status (Supplementary Table 1) were used to generate a wealth index using a principal component analysis (PCA) [10]. Variables were included in the PCA unless >95% of the participating families, or <5%, owned the asset to ensure a reasonable level of variation was included [10]. The first principal component of the PCA was extracted to form the wealth index, then divided into equal wealth quintiles.

The presence/absence of *S mansoni* was determined by a urine schistosome circulating cathodic antigen (CCA) rapid diagnostic test. The intensity of an *S mansoni* infection was inferred from Kato–Katz fecal egg counts as eggs per gram of feces and split into low (<100), moderate (100–399), and high (>399) intensity according to World Health Organization guidelines [11]. Parasitemia/µL blood, assessed from Giemsa-stained blood films, was used to determine both presence/absence and intensity of *Plasmodium* infection. Presence of STHs was determined using the Kato–Katz method. Praziquantel and albendazole were provided to all children at the baseline survey, regardless of infection status in line with mass drug administration protocols. Antimalarials (Lonart) were provided if *Plasmodium* was detected by microscopy or by a fingerpick rapid diagnostic test [9].

Of the 1211 children in the original dataset, 520 were excluded due to an incomplete sample profile (Supplementary Figure 1). Data from missing participants were representative of those individuals included for all variables in the analyses (Supplementary Table 2). The remaining 706 children provided a blood sample for *Plasmodium* diagnosis, a urine sample for schistosome CCA, and a stool sample to detect schistosome and STH egg patent infections, at both baseline and the 6-month survey. The participants’ summary statistics are included in Supplementary Table 3.

All analyses were completed in R version 3.5.2 statistical software (R Core Team, 2018). The effect of infection history (infection at baseline) and coinfection (coinfection at the 6-month survey) on (1) *Plasmodium* intensity, log transformed to normalize the distribution (Ln[x + 1]), (2) *Plasmodium* risk, and (3) *S mansoni* risk were assessed in a series of general linear (*Plasmodium* intensity) and generalized linear (risk models) mixed models using the ASReml-R v4 mixed modeling package (VSN International Ltd, Hemel Hempstead, United Kingdom). In each assessment 2 models were created, with one set incorporating all infections as presence/absence data and the other including *S mansoni* and *Plasmodium* (Ln[x + 1]) data as inferred intensities. These latter models allowed the investigation of potential nonlinear relationships between the parasites. *Plasmodium* intensity models had a Gaussian error distribution and an identity link function. Risk models used a binomial error distribution with a logit link function.

Baseline prevalence of STH was included in all models; however, the 6-month follow-up STH prevalence was only 3.9% and therefore excluded. All starting models included a child’s age, sex, family’s wealth quintile, tribe, and mother’s occupation and education level; behavioral variables associated with *S mansoni* infection (the number of times a child bathed and how long they spent in water) and *Plasmodium* infection (the use of bed nets, the presence of mosquitoes in the home, and whether a person sleeps outside); and the random terms of family identifier, the child’s village, nested within lake, and age fitted with a cubic smoothing spline, as parasite-age profiles are often nonlinear [12]. Detailed model structures can be found in Supplementary Table 3. For all models, the random terms were refined using maximum likelihood ratio tests, then stepwise deletion of insignificant fixed terms was conducted using the Wald test and assessment of the F-statistic (significance limit taken as *P* = .05).

## RESULTS

**Figure 1.**
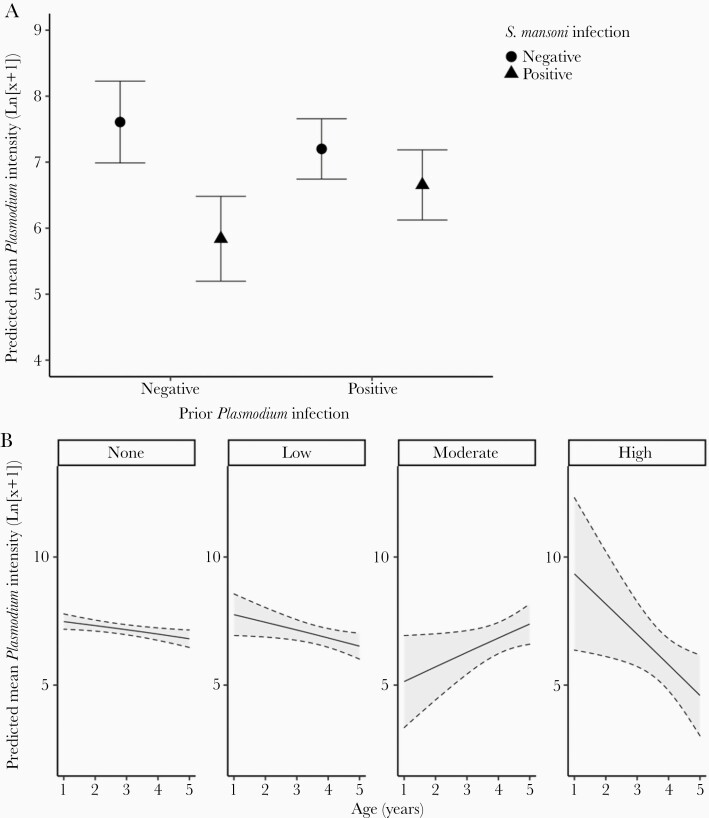
The change in the predicted mean *Plasmodium* intensity (Ln[x+1]) at the 6-month survey in relation to *Schistosoma mansoni* coinfection, with and without prior *Plasmodium* infection (*A*), and prior *S mansoni* infection intensity with child age (*B*). *A*, Children were either uninfected with *S mansoni* at the 6-month survey (circles) or infected (triangles). Predictions were made with age at the median (3 years), sex set to male, and the wealth quintile set to 3. *B*, None, low, moderate, and high represent the intensity of the prior *S mansoni* infection. Age and a prior *S mansoni* infection were the only significant terms. Error bars or dotted lines represent 95% confidence intervals for the predictions.

Baseline *Plasmodium* infection (presence/absence), in interaction with an *S mansoni* coinfection, was associated with a significant reduction in intensity of *Plasmodium* (*P =* .001; Figure 1A). For children with no prior history of *Plasmodium*, current coinfection with *S mansoni* was associated with a lower intensity of *Plasmodium* infection (*P < *.001; Table 1). In children who had a baseline *Plasmodium* infection, there was no significant association with a current *S mansoni* coinfection (*P = *.126; Table 1). In *S mansoni–*coinfected children, *Plasmodium* infection intensity had a U-shaped relationship with wealth, with significant differences at moderate wealth quintiles (3–4; Table 1).

**Table 1.** Generalized Linear Mixed-Model Analyses of the Relationship Between *Plasmodium* Intensity (Ln[x+1]) and *Schistosoma mansoni*, Prior *Plasmodium* Infection (Presence/Absence), and Wealth; and Prior *S mansoni* Eggs per Gram of Feces and Age of 520 Preschool-Aged Children in Uganda, 2009–2011

Variable	Group	Estimate	SE	95% CI	F-Statistic	*P* Value
Presence/Absence of prior infections and *S mansoni* coinfection on *Plasmodium* intensity						
* S mansoni*/ prior *Plasmodium*	*S mansoni* neg/ *Plasmodium* neg	7.60	0.32	6.97–8.24	*F*_*1506*_ *=* 9.2	.001*
	*S mansoni* pos/ *Plasmodium* neg	–1.77	0.45	5.18–6.49		<.001*
	*S mansoni* neg/ *Plasmodium* pos	–0.41	0.27	6.71–7.68		.128
	*S mansoni* pos/ *Plasmodium* pos	–0.95	0.42	6.10–7.20		.022*
* S mansoni/* wealth quintile	*S mansoni* neg/1	7.34	0.31	6.72–7.96	*F*_*4506*_ *=* 3	.018*
	*S mansoni* neg/2	0.37	0.39	7.00–8.41		.351
	*S mansoni* neg/3	0.26	0.35	6.97–8.24		.455
	*S mansoni* neg/4	0.66	0.35	7.37–8.62		.064
	*S mansoni* neg/5	–0.26	0.36	6.44–7.72		.474
	*S mansoni* pos/1	–0.36	0.42	6.38–7.58		.398
	*S mansoni* pos/2	–0.97	0.42	5.79–6.95		.022*
	*S mansoni* pos/3	–1.41	0.44	5.17–6.49		.001*
	*S mansoni* pos/4	–1.19	0.45	5.48–6.83		.009*
	*S mansoni* pos/5	–1.14	0.50	5.40–7.00		.025*
* *Family ID	…	0.27	0.29	…		
* *Age (fitted with a spline curve)	…	0.0003	0.006	…		
* *Residual variation	…	3.01	0.33	…		
Inferred intensities of prior infections and *S mansoni* coinfection on *Plasmodium* intensity						
* *Prior *S mansoni* EPG/age (years)	None/1	7.48	0.15	7.18–7.78	*F*_*3511*_ *=* 3.8	.011*
	None/median	–0.32	0.10	6.96–7.36		.023*
	None/5	–0.67	0.17	6.47–7.15		.010*
	Low/1	0.27	0.41	6.94–8.56		.541
	Low/median	–0.33	0.21	6.75–7.55		.203
	Low/5	–0.96	0.26	6.02–7.02		.001*
	Moderate/1	–2.34	0.92	3.34–6.93		.012*
	Moderate/median	–1.20	0.43	5.43–7.13		.009*
	Moderate/5	–0.10	0.40	6.60–8.17		.827
	High/1	1.86	1.51	6.36–12.32		.223
	High/median	–0.49	0.65	5.72–8.25		.458
	High/5	–2.88	0.81	3.02–6.18		<.001*
Family ID	…	0.09	0.29	…		
Age (fitted with a spline curve)	…	<0.0001	0.006	…		
Residual variation	…	3.11	0.35	…		

Significant explanatory variables and groups are denoted by an asterisk (*). Median = median age of preschool-aged children (3 years).

Abbreviations: CI, confidence interval; EPG, eggs per gram of feces; ID, identifier; neg, negative; pos, positive; SE, standard error;.

In the models where infection intensities were considered, the baseline *S mansoni* intensity, in interaction with age, had a nonlinear relationship with *Plasmodium* infection intensity (*P =* .011; Figure 1B). *Plasmodium* intensity fell as age increased except for children with a moderate (n = 34) *S mansoni* infection, whereas *Plasmodium* intensity increased with age. The age-related decline in *Plasmodium* intensity was steeper for those children with prior low-intensity (n = 101) and high-intensity (n = 13) *S mansoni* infections. In children with moderate baseline infections, although *Plasmodium* intensity increased with age, it remained lower than in either infection category for the equivalent age group. Cumulative *Plasmodium* intensity is therefore lowest in children with moderate prior *S mansoni* infection (Supplementary Figure 3).

Coinfection was not associated with a change in infection risk of either parasite; however, each species was associated with an increase in its own subsequent risk of infection (Supplementary Results). Age and sex were also significantly associated with changes in *Plasmodium* and *S mansoni* risk (Supplementary Results).

## DISCUSSION

In our analysis we detected, for the first time, an association between *Plasmodium* and *S mansoni* in preschool-aged children. In contrast to our hypothesis, we see that coinfection with *S mansoni* and *Plasmodium* is associated with a reduced *Plasmodium* infection intensity. This relationship between *Plasmodium* and *S mansoni* is, however, complex and moderated by host age, family wealth, and prior infections. There was no apparent effect of *S mansoni* on the risk of *Plasmodium* infection nor *Plasmodium* on the risk of *S mansoni*.

The body’s response to *S mansoni* infection is generally considered to be biased toward a T-helper 2 (Th2) response that is largely directed against schistosome eggs [13]. As the Th2 response is co-downregulatory to the T-helper 1 (Th1) response, the primary response required for *Plasmodium* clearance [13, 14], it might be expected that coinfection would lead to increased *Plasmodium* intensity, whereas we observed a reduction. Early-stage schistosome infections (ie, before egg production), however, stimulate a Th1 response [13]. Of the preschool-aged children in our study, only 29% had egg patent infections, suggesting that many *S mansoni* infections may have been in the Th1 phase of immune stimulation. An early *S mansoni*–induced Th1 response could negatively impact *Plasmodium* and explain the reduced intensity of *Plasmodium* we observe. Nevertheless, there is the possibility that the relationship between these parasite species is driven by resource competition. *Plasmodium* species and hookworms have been observed to compete for the same resource, blood, with hookworm coinfection reducing *Plasmodium* intensity [15]. Blood is also a resource for schistosomes; however, resource competition would not explain the increased *Plasmodium* intensity seen in schistosome coinfections in older children [4].

When we explored the relationship between *Plasmodium* and prior *S mansoni* infection in respect to different schistosome intensities, a child’s age was an important moderator of this relationship. Age was associated with lower *Plasmodium* intensity at low and high prior *S mansoni* intensities; however, *Plasmodium* intensity increased with age for moderate infections. Nonlinear effects of schistosomes on *Plasmodium* have been observed elsewhere, with moderate schistosome infections reducing the incidence of *Plasmodium* infection while high- and low-intensity infections are associated with increased incidence [5]. It is feasible that the interactions between these parasites is both immune and resource mediated, and it may be that the interplay between these 2 mechanisms varies, with the effect of one becoming more dominant than the other at different stages of infection. The low number of high-intensity *S mansoni* infections is a limitation of our data. In older children most studies have observed positive associations between schistosome infection and *Plasmodium* intensity [4, 6]. This shift to a positive association could be observed if more children of this age have moderate infection intensities, as the child age–*Plasmodium* intensity trajectory we observe is positive.

The SIMI data do not contain complete information on all *Plasmodium* species present. Three species of *Plasmodium* can be found within the studied communities, but *P falciparum* dominates (75%) [11]. One study has reported differential outcomes of coinfection between hookworms and different *Plasmodium* species [15], and further work should assess whether our findings are robust to all *Plasmodium* species.

Assessing infections in preschool-aged children with known infection histories has enabled us to elucidate the complex relationship between *Plasmodium* and *S mansoni*. We show that prior infections can have long-lasting effects and suggest that *Plasmodium*–schistosome interactions are likely to be mediated by the host immune response since any infections have been removed or reduced below detection limits by chemotherapeutic interventions. Determining the order of infection was essential to understanding coinfection outcomes in this system. Our results also suggest that stage of infection (early or later-stage) may have an important influence on the relationship between *Plasmodium* and schistosomes, again likely mediated by the host immune response. Our work has focused on the effect parasites have on one another, but it will now be important to identify whether, and how, these interactions affect host health and the efficacy of the individual parasite control strategies, highlighting the potential need for an integrated *Plasmodium*–schistosome control strategy for this more vulnerable age group.

## Supplementary Data

Supplementary materials are available at *The Journal of Infectious Diseases* online. Supplementary materials consist of data provided by the author that are published to benefit the reader. The posted materials are not copyedited. The contents of all supplementary data are the sole responsibility of the authors. Questions or messages regarding errors should be addressed to the author.

jiac072_suppl_Supplementary_Figure_S1

jiac072_suppl_Supplementary_Figure_S2

jiac072_suppl_Supplementary_Figure_S3

jiac072_suppl_Supplementary_Figure_S4

jiac072_suppl_Supplementary_Figure_S5

jiac072_suppl_Supplementary_Figure_S6

jiac072_suppl_Supplementary_Figure_S7

jiac072_suppl_Supplementary_Figure_S8

jiac072_suppl_Supplementary_Figure_S9

jiac072_suppl_Supplementary_Table_S1

jiac072_suppl_Supplementary_Table_S2

jiac072_suppl_Supplementary_Table_S3

jiac072_suppl_Supplementary_Table_S4

jiac072_suppl_Supplementary_Table_S5

jiac072_suppl_Supplementary_Table_S6

## Notes

Presented in part: British Ecological Society Annual Meeting, Belfast, Northern Ireland, United Kingdom, 10–13 December 2019; and British Society for Parasitology Conference, Virtual, 21–25 June 2021.

***Acknowledgments.*** We thank the technicians of the Vector Control Division, Uganda Ministry of Health, and the rest of the team behind the Schistosomiasis in Mothers and Infants (SIMI) project for their support in data collection in the field and for assistance with data entry. We are very grateful to all guardians and their children who participated in the SIMI study.

***Financial support.*** This study was supported by Cardiff University’s Wellcome Trust Institutional Strategic Support Fund (number 204824/Z/16/Z): “Untangling intestinal schistosomiasis and malaria interactions using a longitudinal cohort study of mothers and pre-school children in Uganda.”

***Potential conflicts of interest*.** All authors: No reported conflicts of interest.
