# Supplementary material for: Infection History and Current Coinfection With Schistosoma mansoni Decreases Plasmodium Species Intensities in Preschool Children in Uganda
Source: J Infect Dis. 2022 Mar 5;225(12):2181–6. doi: 10.1093/infdis/jiac072 (PMC9200150; doi:10.1093/infdis/jiac072)
Supplement: jiac072_suppl_Supplementary_Figure_S1 [file jiac072_suppl_supplementary_figure_s1.docx]

Supplementary figure 1: Flow chart representing the exclusion criteria for the study. Of the 1211 children enrolled in the study, 706 provided blood, faecal, and urine samples at the baseline and six-month follow up and are eligible for analysing the association of risk factors on *Plasmodium*/*S. mansoni* infection. After excluding children negative for *Plasmodium* presence at the six-month follow up, 520 children were eligible for analysing the association of risk factors on *Plasmodium* intensity.
