# Supplementary material for: Infection History and Current Coinfection With Schistosoma mansoni Decreases Plasmodium Species Intensities in Preschool Children in Uganda
Source: J Infect Dis. 2022 Mar 5;225(12):2181–6. doi: 10.1093/infdis/jiac072 (PMC9200150; doi:10.1093/infdis/jiac072)
Supplement: jiac072_suppl_Supplementary_Figure_S2 [file jiac072_suppl_supplementary_figure_s2.docx]

Supplementary figure 2


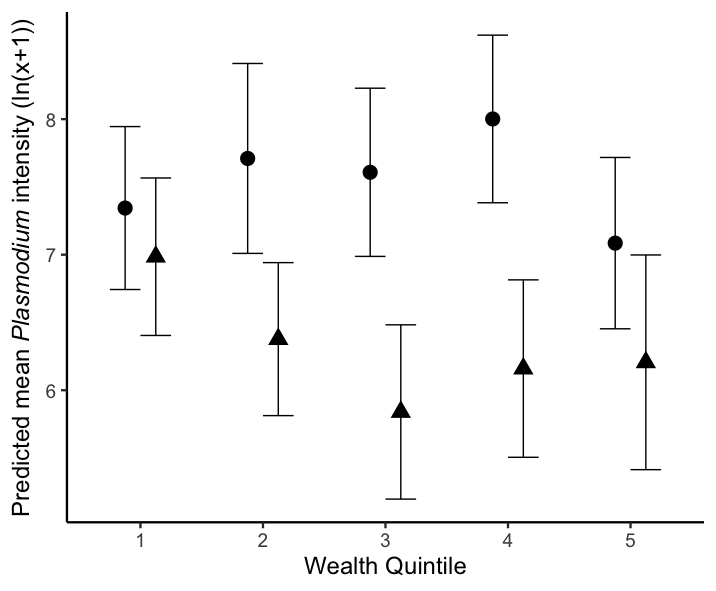


Supplementary figure 2: The change in the predicted mean *Plasmodium* intensity Ln(x+1) at the six-month survey, in relation to *Schistosoma mansoni* coinfection, with wealth quintiles. Children were either uninfected with *S. mansoni* at the six-month survey (circles) or infected (triangles). Predictions were made with age at the median, sex set to male and to have no prior *Plasmodium* infection. Error bars or dotted lines represent 95% confidence intervals for the predictions.
