# Supplementary material for: Infection History and Current Coinfection With Schistosoma mansoni Decreases Plasmodium Species Intensities in Preschool Children in Uganda
Source: J Infect Dis. 2022 Mar 5;225(12):2181–6. doi: 10.1093/infdis/jiac072 (PMC9200150; doi:10.1093/infdis/jiac072)
Supplement: jiac072_suppl_Supplementary_Figure_S3 [file jiac072_suppl_supplementary_figure_s3.docx]

Supplementary Figure 3


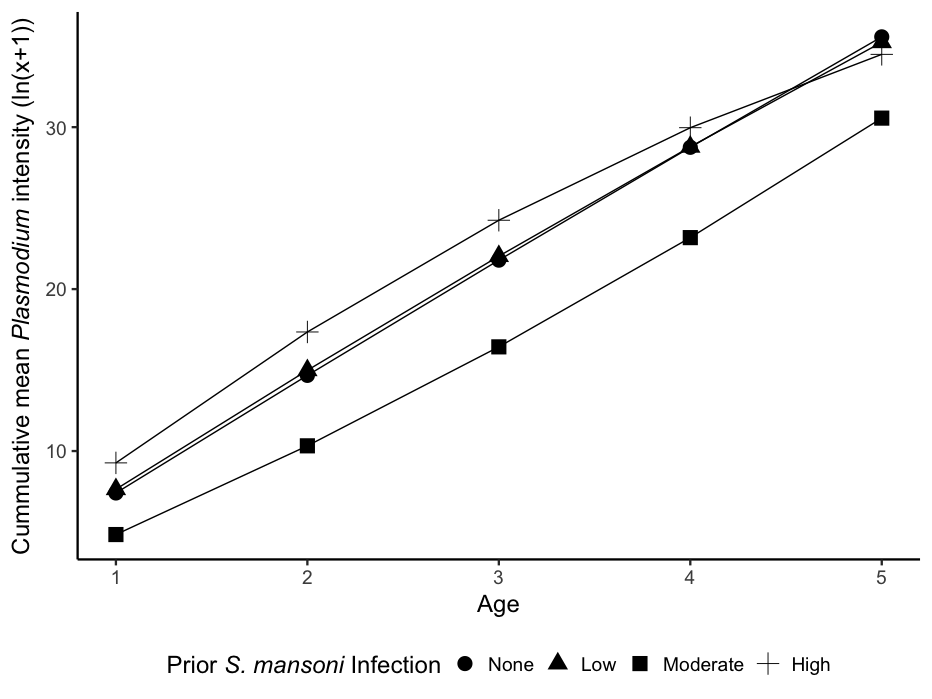


Supplementary Figure 3: The cumulative means of the ln(x+1) predicted *Plasmodium* intensity over a PSAC’s age for None (circles), Low (triangles), moderate (squares) and High (crosshairs) prior *S. mansoni* infection intensities.
