# Supplementary material for: Infection History and Current Coinfection With Schistosoma mansoni Decreases Plasmodium Species Intensities in Preschool Children in Uganda
Source: J Infect Dis. 2022 Mar 5;225(12):2181–6. doi: 10.1093/infdis/jiac072 (PMC9200150; doi:10.1093/infdis/jiac072)
Supplement: jiac072_suppl_Supplementary_Figure_S4 [file jiac072_suppl_supplementary_figure_s4.docx]

Supplementary figure 4


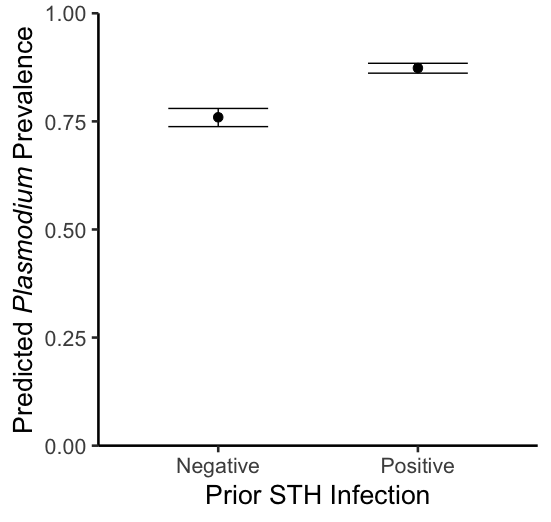


Supplementary Figure 2: The mean *Plasmodium* infection risk predicted for children with and without a prior infection of a soil transmitted helminth. Predictions were made with the wealth quintile set to 3 (moderate). Error bars represent 95% confidence intervals.
