# Supplementary material for: Infection History and Current Coinfection With Schistosoma mansoni Decreases Plasmodium Species Intensities in Preschool Children in Uganda
Source: J Infect Dis. 2022 Mar 5;225(12):2181–6. doi: 10.1093/infdis/jiac072 (PMC9200150; doi:10.1093/infdis/jiac072)
Supplement: jiac072_suppl_Supplementary_Figure_S5 [file jiac072_suppl_supplementary_figure_s5.docx]

Supplementary Figure 5


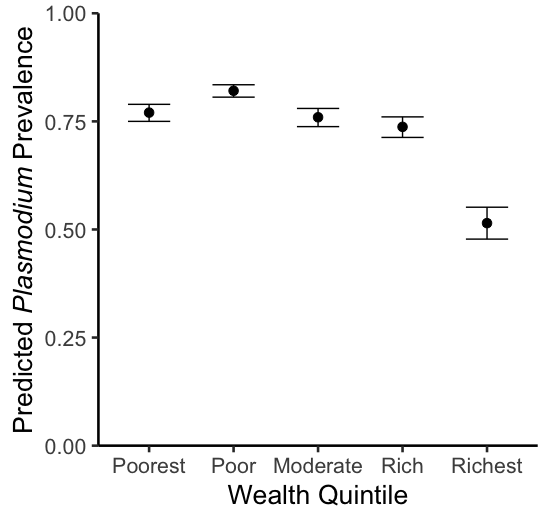


Supplementary Figure 5: The mean *Plasmodium* infection risk predicted for children at different relative wealth quintiles. Predictions were made with the prior soil transmitted helminth infection set to 0 (not infected). Error bars represent 95% confidence intervals.
