# Supplementary material for: Infection History and Current Coinfection With Schistosoma mansoni Decreases Plasmodium Species Intensities in Preschool Children in Uganda
Source: J Infect Dis. 2022 Mar 5;225(12):2181–6. doi: 10.1093/infdis/jiac072 (PMC9200150; doi:10.1093/infdis/jiac072)
Supplement: jiac072_suppl_Supplementary_Figure_S7 [file jiac072_suppl_supplementary_figure_s7.docx]

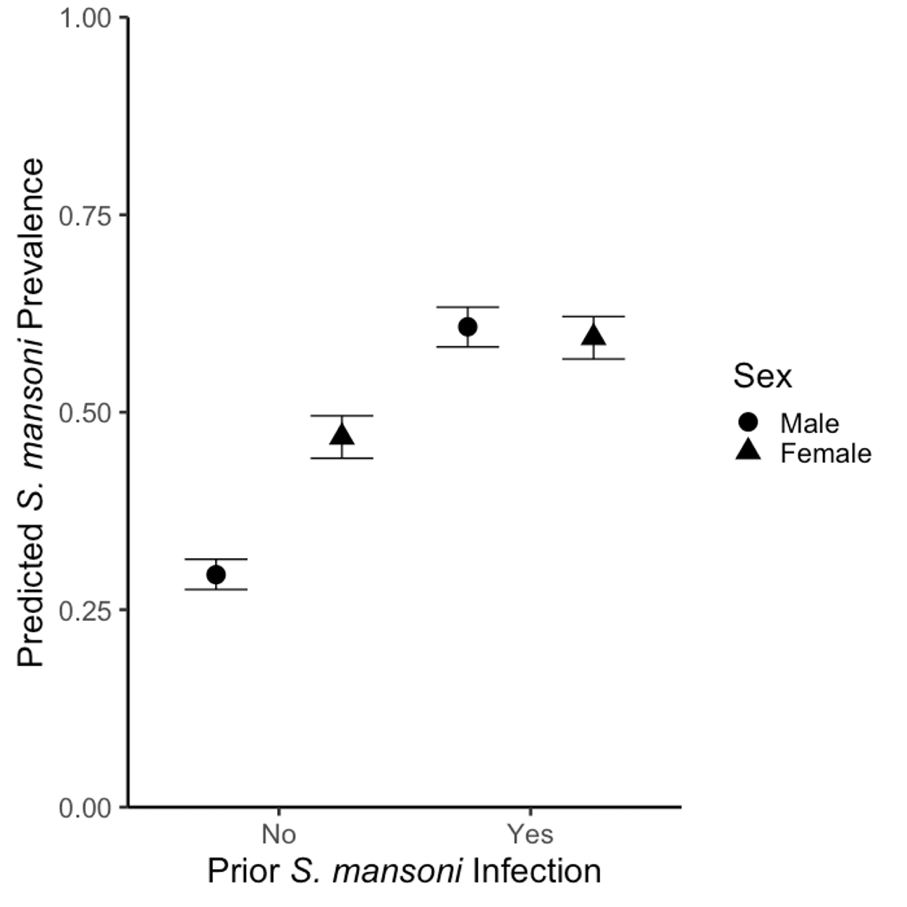
Supplementary Figure 7

Supplementary Figure 7: The mean *Schistosoma mansoni* infection risk predicted for male (Circles) and female (Triangles) children with and without a prior *S. mansoni* infection. Sex and a prior *S. mansoni* infection (presence/absence) were the only significant terms. Error bars represent 95% confidence intervals.
