# Supplementary material for: Infection History and Current Coinfection With Schistosoma mansoni Decreases Plasmodium Species Intensities in Preschool Children in Uganda
Source: J Infect Dis. 2022 Mar 5;225(12):2181–6. doi: 10.1093/infdis/jiac072 (PMC9200150; doi:10.1093/infdis/jiac072)
Supplement: jiac072_suppl_Supplementary_Figure_S8 [file jiac072_suppl_supplementary_figure_s8.docx]

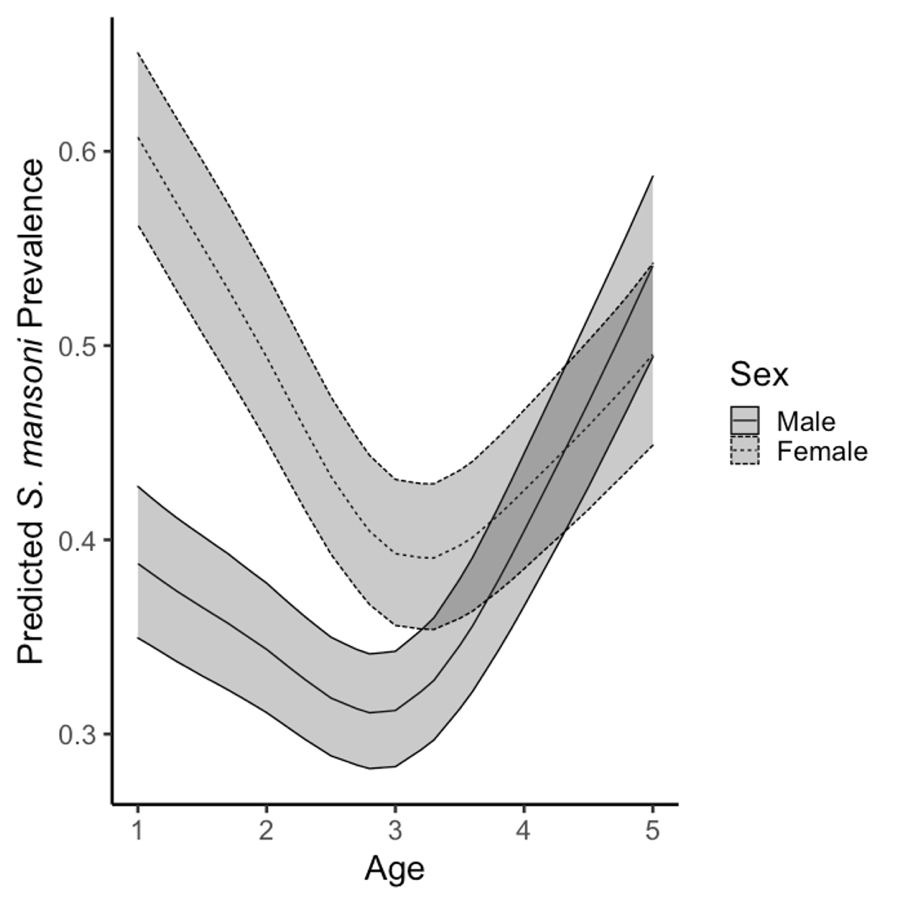
Supplementary Figure 8

Supplementary Figure 8: The mean *Schistosoma mansoni* infection risk predicted for male (block line) and female (dashed line) children over a child’s age. Predictions were made with the prior *S. mansoni* infection intensity set to “none”. Shaded areas represent 95% confidence intervals.
