# Supplementary material for: Infection History and Current Coinfection With Schistosoma mansoni Decreases Plasmodium Species Intensities in Preschool Children in Uganda
Source: J Infect Dis. 2022 Mar 5;225(12):2181–6. doi: 10.1093/infdis/jiac072 (PMC9200150; doi:10.1093/infdis/jiac072)
Supplement: jiac072_suppl_Supplementary_Figure_S9 [file jiac072_suppl_supplementary_figure_s9.docx]

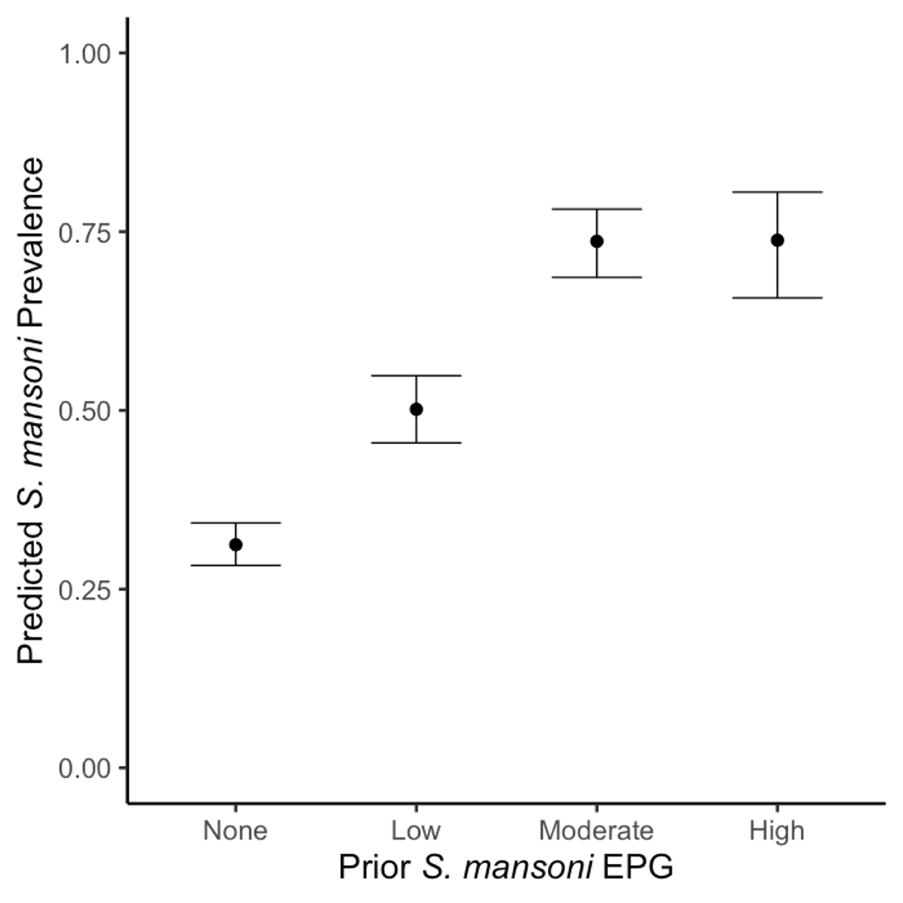
Supplementary Figure 9

Supplementary Figure 9: The mean *Schistosoma mansoni* infection risk predicted for children with different prior *S. mansoni* infection intensities. None, low, moderate and high represent the intensity of the prior *S. mansoni* infection. Predictions were made with sex set to male (0) and age set to the median (3).
