# Supplementary material for: Infection History and Current Coinfection With Schistosoma mansoni Decreases Plasmodium Species Intensities in Preschool Children in Uganda
Source: J Infect Dis. 2022 Mar 5;225(12):2181–6. doi: 10.1093/infdis/jiac072 (PMC9200150; doi:10.1093/infdis/jiac072)
Supplement: jiac072_suppl_Supplementary_Table_S1 [file jiac072_suppl_supplementary_table_s1.docx]

Supplementary Table 1: List of assets, summary statistics and the asset factor score for variables used in the construction of the wealth index – the asset factor score was taken from the first principal component of the Principal Component Analysis (PCA).

| Asset variable | Mean | Standard Deviation | Proportion of people with asset | Asset factor score |
| --- | --- | --- | --- | --- |
| *Asset Ownership* |  |  |  |  |
| Bed | 0.77669903 | 0.41657082 | 77.67 | 0.1868538 |
| Bicycle | 0.32275417 | 0.46765539 | 32.28 | 0.12480332 |
| Canoe | 0.14008621 | 0.34716999 | 14.01 | -0.0743139 |
| Cattle | 0.12406015 | 0.32973872 | 12.41 | 0.11209193 |
| Poultry | 0.40923738 | 0.49182523 | 40.92 | 0.04233256 |
| Goat or Sheep | 0.20817644 | 0.40611295 | 20.82 | 0.15435738 |
| Mobile Phone | 0.34373319 | 0.47508115 | 34.37 | 0.02816912 |
| Radio | 0.50672405 | 0.50008931 | 50.67 | 0.11861589 |
| *Light type* |  |  |  |  |
| None | 0.16367591 | 0.37008186 | 16.37 | 0.17638076 |
| Candle | 0.61663948 | 0.48633718 | 61.66 | -0.3054643 |
| Oil | 0.21968461 | 0.41414557 | 21.97 | 0.12908351 |
| *Stove type* |  |  |  |  |
| None | 0.25537634 | 0.43618982 | 25.54 | 0.25022832 |
| Charcoal | 0.73978495 | 0.43886983 | 73.98 | -0.2572981 |
| *Toilet facility* |  |  |  |  |
| None | 0.0691689 | 0.25380919 | 6.92 | -0.0412972 |
| Communal | 0.11903485 | 0.32391637 | 11.9 | -0.021762 |
| Family | 0.8155496 | 0.38795509 | 81.55 | 0.06677512 |
| *House construction* |  |  |  |  |
| *Floor* |  |  |  |  |
| Mud | 0.86734694 | 0.33929051 | 86.73 | -0.1798063 |
| Cement | 0.10257787 | 0.30348825 | 10.26 | 0.1498192 |
| *Roof* |  |  |  |  |
| Thatched | 0.70426336 | 0.45649638 | 70.43 | -0.4098264 |
| Tin | 0.26497572 | 0.44143941 | 26.50 | 0.35696538 |
| *Walls* |  |  |  |  |
| Mud | 0.72234157 | 0.44796428 | 72.23 | -0.3912513 |
| Grass | 0.06552095 | 0.2475093 | 6.55 | 0.0640372 |
| Brick | 0.21213749 | 0.40893151 | 21.21 | 0.21213749 |
