# Supplementary material for: Infection History and Current Coinfection With Schistosoma mansoni Decreases Plasmodium Species Intensities in Preschool Children in Uganda
Source: J Infect Dis. 2022 Mar 5;225(12):2181–6. doi: 10.1093/infdis/jiac072 (PMC9200150; doi:10.1093/infdis/jiac072)
Supplement: jiac072_suppl_Supplementary_Table_S2 [file jiac072_suppl_supplementary_table_s2.docx]

| Variable  Supplementary table 2: Differences in the number of children included and excluded from the GLMM analyses. Significance is denoted by asterisks *. | Included | Excluded | X^2^ | P value |
| --- | --- | --- | --- | --- |
| Prior *Plasmodium* + | 518 | 305 | 1.84 | 0.17 |
| Prior *Plasmodium* - | 188 | 134 | - | - |
| Prior *Plasmodium* <5000 parasites/µl | 395 | 232 | 2.03 | 0.36 |
| Prior *Plasmodium >*5000 parasites/µl | 132 | 73 | - | - |
| Prior *S. mansoni* + | 349 | 186 | 1.57 | 0.21 |
| Prior *S. mansoni* - | 357 | 160 | - | - |
| Prior low *S. mansoni* EPG | 139 | 90 | 0.30 | 0.96 |
| Prior moderate *S. mansoni* EPG | 45 | 25 | - | - |
| Prior high *S. mansoni* EPG | 16 | 10 | - | - |
| Prior STH + | 90 | 40 | 6.11 | 0.013* |
| Prior STH - | 604 | 404 | - | - |
| *Plasmodium* + | 520 | 92 | <0.001 | 0.99 |
| *Plasmodium* - | 186 | 32 | - | - |
| *Plasmodium* <5000 parasites/µl | 405 | 64 | 3.10 | 0.22 |
| *Plasmodium* >5000 parasites/µl | 115 | 28 | - | - |
| *S. mansoni* + | 347 | 47 | 2.29 | 0.13 |
| *S. mansoni* - | 359 | 33 | - | - |
| Low *S. mansoni* EPG | 89 | 9 | 1.16 | 0.76 |
| Moderate *S. mansoni* EPG | 33 | 4 | - | - |
| High *S. mansoni* EPG | 8 | 1 | - | - |
| **Sex** |  |  |  |  |
| Male | 372 | 215 | 4.42 | 0.036* |
| Female | 334 | 250 | - | - |
| **How often do you bathe?** |  |  |  |  |
| Once per day | 158 | 84 | 2.83 | 0.093 |
| Twice per day | 546 | 378 | - | - |
| **How long do you spend in water per day?** |  |  |  |  |
| Never | 277 | 68 | 12.4 | 0.014* |
| >30 minutes | 231 | 26 | - | - |
| 30-60 minutes | 50 | 11 | - | - |
| 1-2 hours | 109 | 14 | - | - |
| >2 hours | 30 | 5 | - | - |
| **Do you sleep under a bednet?** |  |  |  |  |
| Yes | 358 | 64 | 0.004 | 0.95 |
| No | 335 | 58 | - | - |
| **Do you sleep inside at night?** |  |  |  |  |
| Yes | 457 | 84 | <0.001 | 1 |
| No | 195 | 34 | - | - |
| **Are you bothered by mosquitoes at home?** |  |  |  |  |
| Yes | 612 | 99 | 8.26 | 0.004* |
| No | 88 | 22 | - | - |
