# Supplementary material for: Infection History and Current Coinfection With Schistosoma mansoni Decreases Plasmodium Species Intensities in Preschool Children in Uganda
Source: J Infect Dis. 2022 Mar 5;225(12):2181–6. doi: 10.1093/infdis/jiac072 (PMC9200150; doi:10.1093/infdis/jiac072)
Supplement: jiac072_suppl_Supplementary_Table_S3 [file jiac072_suppl_supplementary_table_s3.docx]

Supplementary Table 3: - The demographic, behavioural and socio-economic characteristics of the 706 children, along with infection prevalence at the baseline and six-month follow up. The overall infection has been broken down into mono and coinfection prevalence and 95 per cent confidence intervals (CI_95_) or the total number (N).

|  | **Baseline**  **Prevalence (CI_95_/N)** | **Six-month follow up Prevalence (CI_95_/N)** |
| --- | --- | --- |
| **Participants with missing samples** | 120 | 505 |
| **Single and Coinfections** |  |  |
| Single *S. mansoni* | 12.13% (10.2-14.3) | 12.6% (10.1-14.7) |
| Single *Plasmodium* spp. | 31.4% (28.7-34.3) | 35.6% (32.6-38.6) |
| Single STH | 0.9% (0.4-1.5) | 0.7% (0.3-1.3) |
| *S. mansoni* + *Plasmodium* spp. | 29.9% (27.1-32.8) | 35.2% (32.2-38.1) |
| *S. mansoni* + STH | 1.1% (0.6-1.8) | 0.28% (0-0.71) |
| *Plasmodium* spp. + STH | 5.9% (4.5-7.3) | 1.7% (0.99-2.5) |
| *S. mansoni* + *Plasmodium* spp. + STH | 6.0% (4.7-7.6) | 1.3% (0.56-1.98) |
| None | 12.0% (10.0-14.0) | 12.7% (10.7-14.8) |
| **Total Infection** |  |  |
| *S. mansoni* | 49.4% (46.3-52.5) | 49.3% (46.2-52.4) |
| *Plasmodium* spp. | 73.4% (70.6-76.1) | 73.7% (71.0-76.4) |
| STH | 14.2% (12.0-16.4) | 3.96% (2.82-5.23) |
| Mean number of *Plasmodium* spp. parasites/µl | 3159 (2963.2-4123.9) | 5318.3 (4143.5-6609.9) |
| ***Plasmodium* spp.** < 5000 intensity/ul | 395 | 405 |
| > 5000 intensity/ul | 123 | 115 |
| ***S. mansoni* EPG** Low | 140 | 89 |
| Moderate | 45 | 33 |
| High | 16 | 8 |
| Mean EPG | 50 | 25.4 |
| **Lake** |  |  |
| Victoria | - | 56.8% (401) |
| Albert | - | 43.2% (305) |
| **Age** |  |  |
| <1 | - | 7.5% (53) |
| 1-2 | - | 11.2% (79) |
| 2-3 | - | 19.4% (137) |
| 3-4 | - | 20.1% (142) |
| 4-5 | - | 19.5% (138) |
| 5 | - | 22.2% (157) |
| **Sex** |  |  |
| Male | - | 52.7% (372) |
| Female | - | 47.3% (334) |
| **Wealth quintile** |  |  |
| 1 | - | 20% (142) |
| 2 | - | 20% (141) |
| 3 | - | 20% (141) |
| 4 | - | 20% (141) |
| 5 | - | 20% (141) |
| **How often do you bathe?** |  |  |
| Once per day | 22.4% (158) | 22.4% (158) |
| Twice per day | 77.3% (546) | 77.3% (546) |
| **How long do you spend in water per day?** |  |  |
| Never | 36.5% (258) | 39.2% (277) |
| >30 minutes | 27.1% (191) | 32.7% (231) |
| 30-60 minutes | 23.5% (166) | 7.1% (50) |
| 1-2 hours | 7.5% (53) | 15.4% (109) |
| >2 hours | 2.1% (15) | 4.2% (30) |
| **Do you sleep under a bednet?** |  |  |
| Yes | 19.8% (140) | 50.7% (358) |
| No | 64% (452) | 47.5% (335) |
| **Do you sleep inside at night?** |  |  |
| Yes | 54.4% (384) | 64.7% (457) |
| No | 44.5% (314) | 27.6% (195) |
| **Are you bothered by mosquitoes at home?** |  |  |
| Yes | 94.3% (666) | 86.7% (612) |
| No | 5.4% (38) | 12.5% (88) |
