# Supplementary material for: Infection History and Current Coinfection With Schistosoma mansoni Decreases Plasmodium Species Intensities in Preschool Children in Uganda
Source: J Infect Dis. 2022 Mar 5;225(12):2181–6. doi: 10.1093/infdis/jiac072 (PMC9200150; doi:10.1093/infdis/jiac072)
Supplement: jiac072_suppl_Supplementary_Table_S4 [file jiac072_suppl_supplementary_table_s4.docx]

Supplementary Table 4: Detailed GLMM structure for *Plasmodium* infection risk, *Plasmodium* intensity and *Schistosoma mansoni* risk including the fixed and random variables for each model. Each dependent variable had two models, one model included infection variables as presence/absence data, the second model included infection variables as intensity values.

|  | Dependent Variable | | | | | |
| --- | --- | --- | --- | --- | --- | --- |
| Fixed Variable | ***Plasmodium* prevalence** | ***Plasmodium* prevalence** | **log+1 *Plasmodium* GIEMSA** | **log+1 *Plasmodium* GIEMSA** | ***S. mansoni* prevalence** | ***S. mansoni* prevalence** |
| *Infection Variables* |  |  |  |  |  |  |
| *Plasmodium* presence | - | - | - | - | X | - |
| Log+1 *Plasmodium* GIEMSA |  | - |  | - | - | X |
| *S. mansoni* presence | X | - | X | - | - | - |
| *S. mansoni* EPG (Low, Moderate, High) | - | X | - | X | - | - |
| Baseline *Plasmodium* presence | X | - | X | - | X | - |
| Log+1 Baseline *Plasmodium* GIEMSA | - | X | - | X | - | X |
| Baseline *S. mansoni* presence | X | - | X | - | X | - |
| Baseline *S. mansoni* EPG (Low, Moderate, High) | - | X | - | X | - | - |
| Baseline STH presence | X | X | X | X | X | X |
| *Demographic Variables* |  |  |  |  |  |  |
| Tribe | X | X | X | X | X | X |
| Sex | X | X | X | X | X | X |
| Age | X | X | X | X | X | X |
| Wealth quintile | X | X | X | X | X | X |
| Mothers’ education | X | X | X | X | X | X |
| Mothers’ occupation | X | X | X | X | X | X |
| *Plasmodium specific risk factors* |  |  |  |  |  |  |
| Do you sleep inside at night? | X | X | X | X | - | - |
| Do you sleep under a bednet? | X | X | X | X | - | - |
| Do mosquitoes bother you at home? | X | X | X | X | - | - |
| *S. mansoni* *specific risk factors* |  |  |  |  |  |  |
| How long do you spend in the water per day? | - | - | - | - | X | X |
| How many times do you bathe per day? | - | - | - | - | X | X |
| Random Variable |  |  |  |  |  |  |
| Family ID | X | X | X | X | X | X |
| Age (Spline) | X | X | X | X | X | X |
| Lake/Village (Village nested in Lake) | X | X | X | X | X | X |
