# Supplementary material for: Infection History and Current Coinfection With Schistosoma mansoni Decreases Plasmodium Species Intensities in Preschool Children in Uganda
Source: J Infect Dis. 2022 Mar 5;225(12):2181–6. doi: 10.1093/infdis/jiac072 (PMC9200150; doi:10.1093/infdis/jiac072)
Supplement: jiac072_suppl_Supplementary_Table_S5 [file jiac072_suppl_supplementary_table_s5.docx]

| Variable | Group | Estimate | Std. Error | CI | F-statistic | P value |
| --- | --- | --- | --- | --- | --- | --- |
| Prior STH | STH - | 0.76 | 0.06 | 0.74-0.78 | *F*_1, 699_*=5.5* | 0.016* |
|  | STH + | 0.11 | 0.05 | 0.86-0.88 | - | 0.016* |
| Wealth | 1 | 0.77 | 0.06 | 0.75-0.79 | *F*_4,317_*=5.2* | < 0.001* |
|  | 2 | 0.05 | 0.05 | 0.8-0.83 | - | 0.306 |
|  | 3 | -0.01 | 0.06 | 0.74-0.78 | - | 0.853 |
|  | 4 | -0.03 | 0.06 | 0.71-0.76 | - | 0.587 |
|  | 5 | -0.25 | 0.08 | 0.48-0.55 | - | < 0.001* |
| Prior *Plasmodium* infection intensity | 0 | 0.69 | 0.23 | 0.65-0.72 | *F*_1, 696_*=9.6* | 0.008* |
|  | Mean | 0.07 | 0.07 | 0.74-0.78 | - | 0.008* |
|  | Maximum | 0.14 | 0.06 | 0.82-0.85 | - | 0.008* |
| Family-Family variation |  | 0.26 | 0.06 |  |  |  |
| Village |  | 0.23 | 0.19 |  |  |  |
| Residual Variation |  | 1 |  |  |  |  |

Supplementary table 5: GLMM analysis of the relationship between *Plasmodium* risk and a Prior STH infection (presence/absence), wealth and a Prior *Plasmodium* intensity (Ln(X+1)) of 706 preschool-aged children in Uganda 2009-2011. Significant explanatory variables and their groups are denoted by asterisks *. Std. Error = Standard error and CI_95_ = 95% confidence intervals.
