# Supplementary material for: Infection History and Current Coinfection With Schistosoma mansoni Decreases Plasmodium Species Intensities in Preschool Children in Uganda
Source: J Infect Dis. 2022 Mar 5;225(12):2181–6. doi: 10.1093/infdis/jiac072 (PMC9200150; doi:10.1093/infdis/jiac072)
Supplement: jiac072_suppl_Supplementary_Table_S6 [file jiac072_suppl_supplementary_table_s6.docx]

| Variable | Group | Estimate | Std. Error | CI | F-statistic | P value |
| --- | --- | --- | --- | --- | --- | --- |
| a) |  |  |  |  |  |  |
| Prior *S. mansoni* infection : Sex | *S. mansoni* -:Male | 0.23 | 0.07 | 0.21-0.25 | *F_1, 691_=4.2* | 0.023* |
|  | *S. mansoni* + : Male | 0.32 | 0.10 | 0.50-0.60 | - | <0.001* |
|  | *S. mansoni* -:Female | 0.17 | 0.09 | 0.36-0.45 | - | 0.001* |
|  | *S. mansoni* + : Female | 0.33 | 0.11 | 0.51-0.61 | - | <0.001* |
| Family-Family variation |  | 0.29 | 0.22 |  |  |  |
| Age (Fitted with a spline curve) |  | 0.05 | 0.07 |  |  |  |
| Village |  | 0.80 | 0.54 |  |  |  |
| Residual Variation |  | 1 |  |  |  |  |
|  |  |  |  |  |  |  |
| b) |  |  |  |  |  |  |
| Age : Sex | 1 : Male | 0.39 | 0.08 | 0.35-0.43 | *F_1, 701_=5.3* | 0.022* |
|  | 3 : Male | -0.08 | 0.07 | 0.28-0.34 | - | 0.181 |
|  | 5 : Male | 0.15 | 0.10 | 0.49-0.59 | - | 0.058 |
|  | 1 : Female | 0.22 | 0.09 | 0.56-0.65 | - | 0.002* |
|  | 3 : Female | 0.00 | 0.08 | 0.36-0.43 | - | 0.942 |
|  | 5 : Female | 0.11 | 0.10 | 0.45-0.54 | - | 0.139 |
| Prior *S. mansoni* EPG | None | 0.31 | 0.07 | 0.28-0.34 | *F_3, 701_=8.9* | <0.001* |
|  | Low | 0.19 | 0.10 | 0.45-0.55 | - | <0.001* |
|  | Moderate | 0.43 | 0.13 | 0.69-0.78 | - | <0.001* |
|  | High | 0.43 | 0.20 | 0.66-0.81 | - | 0.025* |
| Family-Family variation |  | 0.17 | 0.21 |  |  |  |
| Age (Fitted with a spline curve) |  | 0.07 | 0.09 |  |  |  |
| Village |  | 0.56 | 0.39 |  |  |  |
| Residual Variation |  | 1 |  |  |  |  |

Supplementary Table 6: GLMM analysis of the relationship between *S. mansoni* risk and a) Prior *S. mansoni* (presence/absence)-Sex interaction and b) Age-sex interaction and Prior *S. mansoni* EPG. Significant explanatory variables and their groups are denoted by asterisks *. Std. Error = standard error and CI_95_ = 95% confidence intervals.

Supplementary table 5:Relationship between *S. mansoni* infection risk and a) prior *S. mansoni* infection, Sex and b) Age, sex and prior *S. mansoni* EPG of 706 preschool-aged children in Uganda 2009-2011.

P value is between interacting group terms.
